# A new transitional therizinosaurian theropod from the Early Cretaceous Jehol Biota of China

**DOI:** 10.1038/s41598-019-41560-z

**Published:** 2019-03-22

**Authors:** Xi Yao, Chun-Chi Liao, Corwin Sullivan, Xing Xu

**Affiliations:** 1 Key Laboratory of Vertebrate Evolution and Human Origins of Chinese Academy of Sciences, Institute of Vertebrate Paleontology and Paleoanthropology, Chinese Academy of Sciences, Beijing, China; 2 CAS Center of Excellence in Life and Paleoenvironment, Beijing, China; 3 University of Chinese Academy of Sciences, Beijing, China; 4 Department of Biological Sciences, University of Alberta, Edmonton, Canada; 5 Philip J. Currie Dinosaur Museum, Wembley, Canada

## Abstract

Therizinosaurian theropods evolved many highly specialized osteological features in association with their bulky proportions, which were unusual in the context of the generally gracile Theropoda. Here we report a new therizinosaur, *Lingyuanosaurus sihedangensis* gen. et sp. nov., based on a specimen recovered from the Lower Cretaceous Jehol Group of Lingyuan, Liaoning Province, China, which displays a combination of plesiomorphic and derived features. Most notably, the specimen is characterized by posterior dorsal vertebrae with a complex and unusual laminar structure; an ilium with a highly dorsoventrally expanded preacetabular process showing only slight lateral flaring of the ventral margin, a strongly anterodorsally inclined iliac blade, a small postacetabular process with a strongly concave dorsal margin, and a relatively robust pubic peduncle with a posteroventrally facing distal articular surface; a straight and robust femur with a small lesser trochanter; and a tibia that is longer than the femur. Phylogenetic analysis places *Lingyuanosaurus* in an intermediate position within Therizinosauria, i.e., between the early-branching therizinosaurs such as *Falcarius*, *Jianchangosaurus*, and *Beipiaosaurus* and the late-branching ones such as *Alxasaurus* and *Therizinosaurus*. This new therizinosaur sheds additional light on the evolution of major therizinosaurian characteristics, including particularly the distinctive pelvic girdle and hindlimb morphology seen in this group.

## Introduction

Therizinosauria is a highly specialized theropod clade displaying a number of distinctive features suggestive of a herbivorous diet, including a rostral beak, numerous small leaf-shaped cheek teeth, an elongated neck and a broad pelvis. Therizinosaurs have been recovered mainly from the Cretaceous of Asia and North America, although some putative material has been reported from the Cretaceous of Europe and Africa. There is also one possible therizinosaur specimen from the Lower Jurassic of China^1,2^.

Three definite early-branching therizinosaurs (i.e. non-therizinosaurid therizinosaurs) have been found in China, namely *Alxasaurus elesitaiensis* from the Lower Cretaceous of Nei Mongol (Inner Mongolia) in northern China and *Jianchangosaurus yixianensis* and *Beipiaosaurus inexpectus* from the Lower Cretaceous of Liaoning Province in northeastern China^3–5^. In the present paper, we report a new basal therizinosaur based on a specimen recovered from the Lower Cretaceous Jehol Group of Sihedang, Lingyuan, Liaoning, and discuss its implications for the early evolution of the group. We identify the three maniraptoran manual digits as II-III-IV, following the numbering applied to the wing digits of living theropods in most ornithological literature and some recent paleontological studies^6,7^.

## Results

**Systematic Paleontology**

Theropoda Marsh^8^

Coelurosauria Huene^9^

Therizinosauria Russell, *et al*.^10^

*Lingyuanosaurus sihedangensis* gen. et sp. nov.

Holotype: IVPP (Institute of Vertebrate Paleontology and Paleoanthropology, Chinese Academy of Sciences) V 23589, a disarticulated but associated partial skeleton, including one cervical centrum, five partial dorsal, sacral and caudal vertebrae, one complete caudal vertebra, several dorsal ribs, proximal half of left humerus, distal portion of right humerus, two manual unguals, nearly complete left ilium,?proximal portion of left ischium, complete right femur, incomplete left tibia,?partial right astragalus, and several unidentifiable fragments.

Etymology: The binomial name refers to the fact that the type locality is situated in the town of Sihedang within the county-level city of Lingyuan.

Horizon and Locality: Jehol Group, Lower Cretaceous^11,12^. Sihedang Locality, Lingyuan City, Liaoning Province, China. The fossil-bearing strata at Sihedang have been assigned to the Yixian Formation in some studies^13,14^, but to the Jiufotang Formation in others^15,16^. Resolving this stratigraphic issue is beyond the scope of the present paper.

Diagnosis: A small therizinosaur distinguished from other therizinosaurs by the following autapomorphies: posterior dorsal vertebrae with prominent paradiapophyseal lamina separating anterior and posterior infradiapophyseal fossae, with ventral part of hyposphene expanding transversely to form intumescence which extends as far as postzygapophysis posteriorly and beyond postzygapophysis laterally, and with centroprezygapophyseal lamina extending anteriorly considerably beyond level of prezygapophysis and fusing with opposite lamina along midline; and ilium with most of dorsal margin strongly convex but posterior part of dorsal margin distinctly concave, and sub-triangular fossa immediately dorsal to pubic peduncle on medial surface.

### Description and comparisons

**Figure 1:**
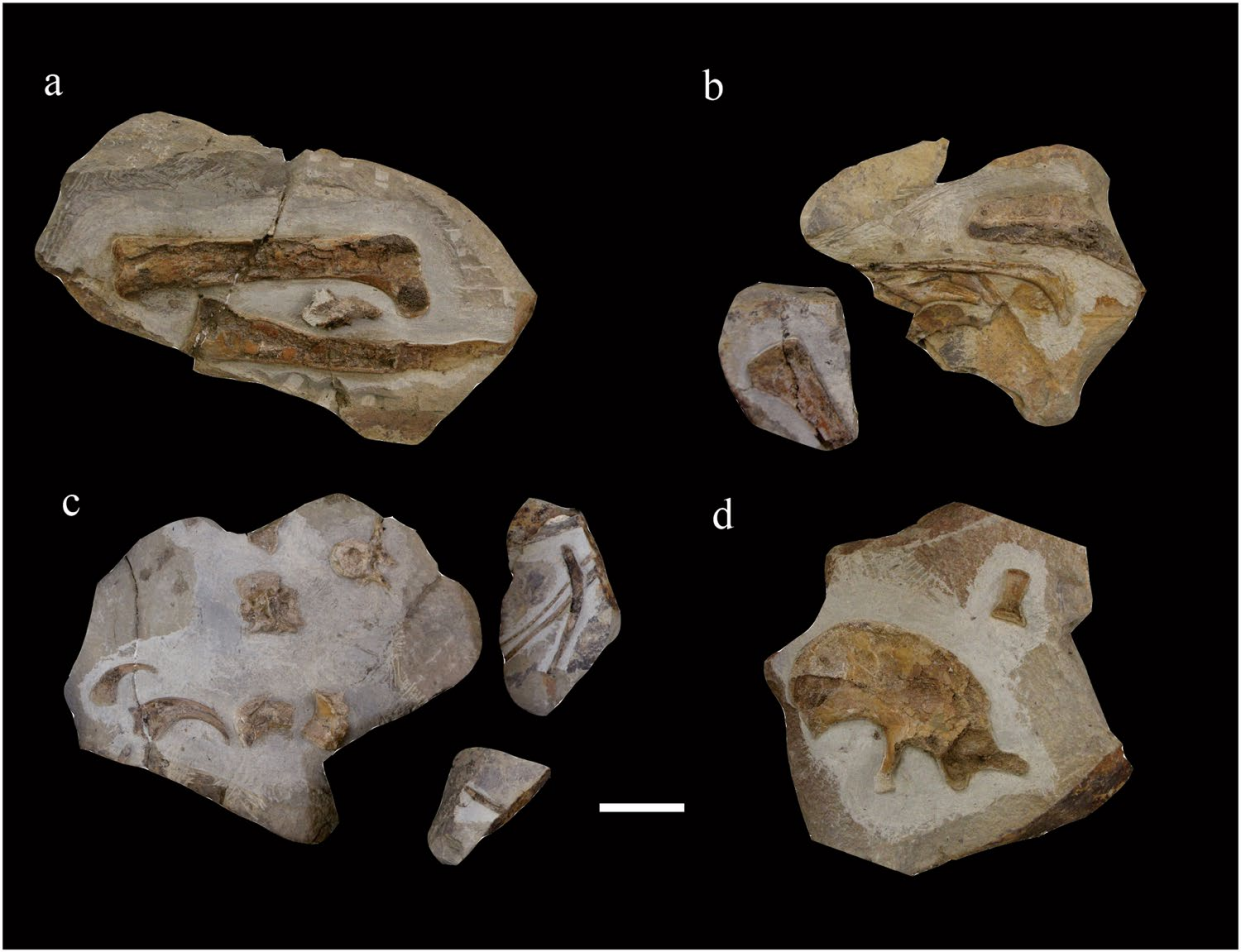
IVPP V 23589 as originally preserved, showing slab preserving right femur and left tibia (**a**); slab preserving ribs, proximal portion of right humerus, and ischium (**b**); slab preserving vertebrae, manual unguals and ribs (**c**); and slab preserving left ilium and right astragalus (**d**). Scale bar = 50 mm.

IVPP V 23589 is represented by a number of postcranial skeletal elements originally preserved in seven slabs of different sizes (Fig. 1), all of which were collected from a small area (less than a square meter) within a single layer of a lacustrine deposit exposed at the Sihedang Locality, Lingyuan City, Liaoning Province, China. Given that there are no other vertebrate fossils nearby, and that none of the preserved skeletal elements are duplicates, we infer that these bones belong to only one individual. This inference is further supported by the consistent morphology and texture of the bones, as well as by the similar lithology of the matrix across all seven slabs. In order to obtain more morphological information, we fully prepared most skeletal elements free of matrix.

With a femoral length of 200 mm, IVPP V 23589 is small, and the living individual is estimated to have had a body mass of 12 kg using an empirical equation applicable to non-avian theropods^17^. IVPP V 23589 is probably a juvenile based on some fusion features: the centra and neural arches of most of the preserved vertebrae are separate, and the sacral vertebrae are not fused to each other. However, the specimen does not represent a hatchling, given that all bones are well ossified and one anterior caudal vertebra displays closed neurocentral sutures.

Seven vertebrae are preserved, including one nearly complete cervical centrum, one nearly complete dorsal neural arch, one nearly complete sacral centrum, one nearly complete anterior caudal vertebra, and three partial vertebrae that are difficult to identify.

**Figure 2:**
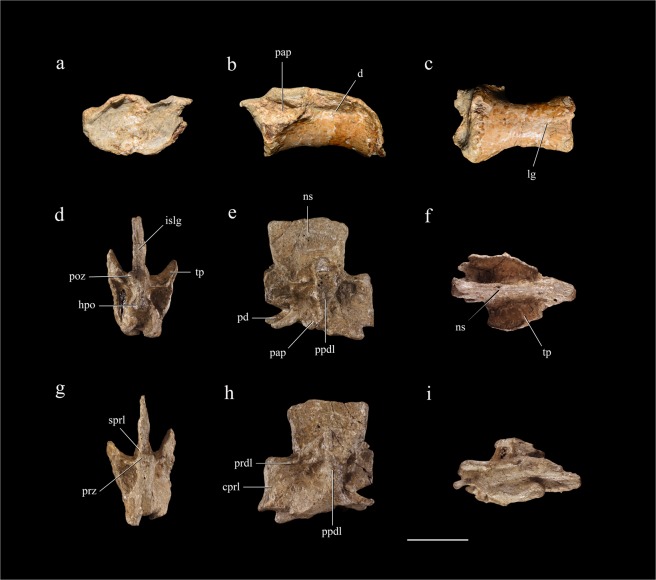
Cervical vertebra and dorsal neural arch. Cervical vertebra in anterior (**a**), left lateral (**b**) and ventral view (**c**); dorsal neural arch in posterior (**d**), right lateral (**e**), dorsal (**f**), anterior (**g**), left lateral (**h**) and ventral view (**i**). Abbreviations: cprl, centroprezygapophyseal lamina; d, depression; hpo, hyposphene; islg, interspinous ligament groove; lg, longitudinal groove; ns, neural spine; pap, parapophysis; pd, pedicle; poz, postzygapophysis; ppdl, paradiapophyseal lamina; prdl, prezygodiapophyseal lamina; prz, prezygapophysis; sprl, spinoprezygapophyseal lamina; tp, transverse process. Scale bar = 20 mm.

The preserved cervical centrum is long (about 30 mm in length) and low in lateral view, the ratio of anteroposterior length to dorsoventral depth (measured at the anterior articular surface) being about 2.0 and the ratio of transverse width to dorsoventral depth being about 1.5. The centrum is slightly procoelous and slightly parallelogram-shaped in lateral view, with the posterior articular surface facing posterodorsally. At the anterodorsal corner of the lateral surface lies a protruding rectangular facet, representing the parapophysis (Fig. 2b). The relatively dorsal position of the parapophysis suggests that this centrum belongs to a posterior cervical vertebra. Posterior to the parapophysis is longitudinal depression, which may represent a pneumatic fossa. There is a weak midline longitudinal groove along the posterior half of the ventral surface of the centrum.

The preserved dorsal neural arch is probably from a posterior dorsal vertebra, as indicated by the position and morphology of the diapophyses and the orientation of the zygapophyses. The neural arch pedicles are short anteroposteriorly, and anteriorly shifted relative to the position of the neural spine. They house a neural canal that is extremely small in diameter. The diapophysis is an anteroposteriorly expanded thin plate, and projects posteriorly, laterally and dorsally. The parapophysis lies directly ventral to the diapophysis, and the two articular structures are connected by a paradiapophyseal lamina. The zygapophyses are small, and the articular facets of the prezygapophyses face nearly medially whereas those of the postzygapophyses face laterally. The hyposphene is prominent, extending as far as the postzygapophyses posteriorly and beyond the postzygapophyses laterally. The walls of the hypantrum are continuous with the prominent centroprezygapophyseal laminae. The posteriorly displaced neural spine is sub-rectangular in lateral view, and the base is slightly shorter anteroposteriorly than the dorsal margin as in *Falcarius* and *Jianchangosaurus*^4,18^. The spine is a transversely thin plate with transversely expanded anterior and posterior margins, the anterior margin being maximally expanded around the middle of its height and the posterior margin being more expanded ventrally than dorsally. The posterior margin bears an interspinous ligament groove which approaches, but fails to reach, the dorsal end of the neural spine. The laminar system is well developed: the paradiapophyseal lamina is prominent and thick, separating distinct anterior and posterior infradiapophyseal fossae (Fig. 2e,h); a thin prezygodiapophyseal lamina connects the lateral margin of the prezygapophysis to the anterior margin of the diapophysis; a low and robust prezygospinous lamina connects the medial margin of the prezygapophysis and the anterior margin of the neural spine; and the centroprezygapophyseal lamina is prominent, expanding anteriorly considerably beyond the level of the prezygapophysis and being situated farther anteriorly than the pedicle. Unusually, the left and right centroprezygapophyseal laminae seem to contact one another anteriorly and fuse together along the midline.

**Figure 3:**
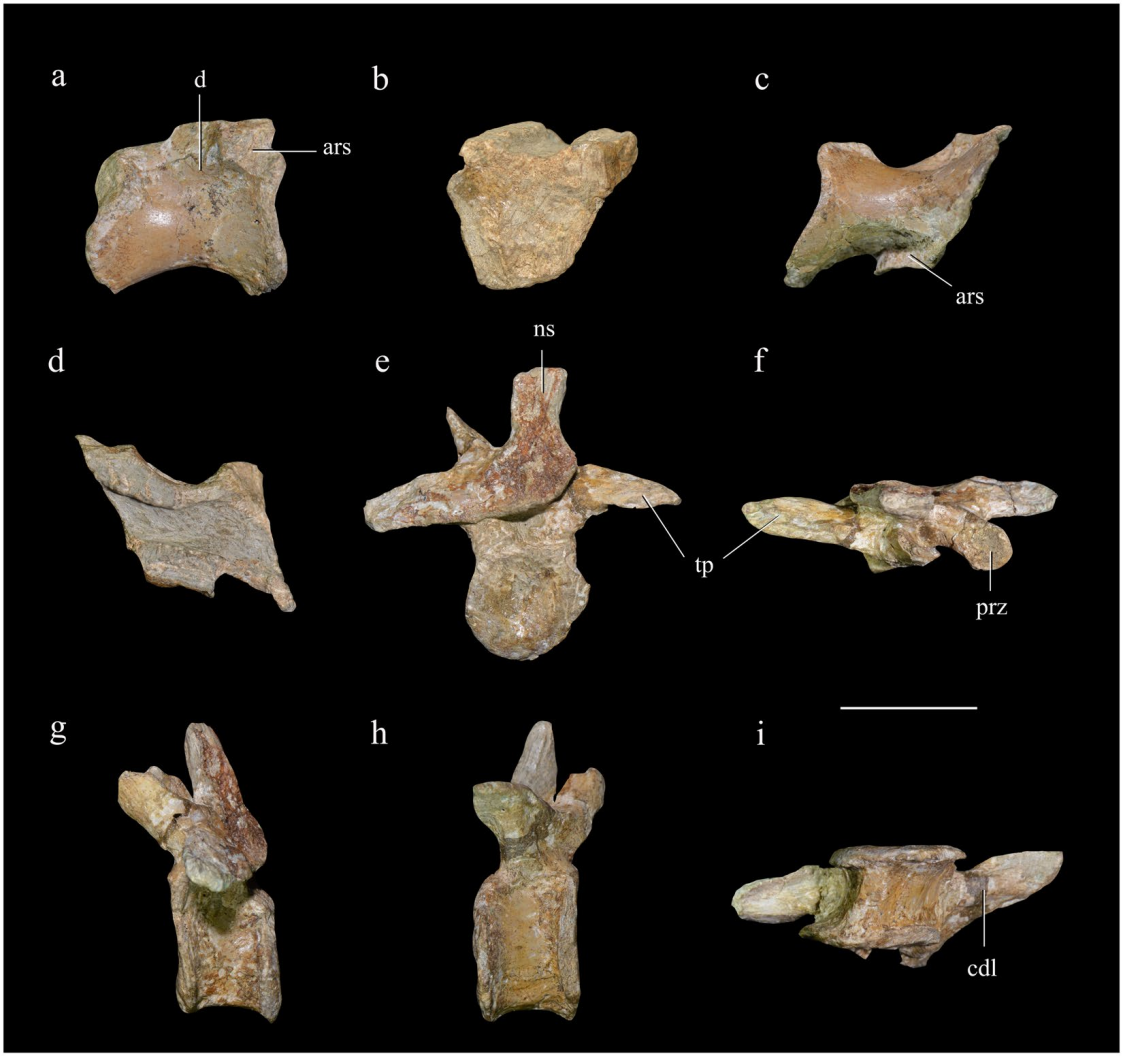
Sacral vertebra and caudal vertebra. Middle sacral centrum in right lateral (**a**), anterior (**b**), ventral (**c**) and dorsal view (**d**); anterior caudal vertebra in posterior (**e**), dorsal (**f**), left lateral (**g**), right lateral (**h**) and ventral view (**i**). Abbreviations: ars, articular surface; cdl, centrodiapophyseal lamina; d, depression; ns, neural spine; prz, prezygapophysis; tp, transverse process. Scale bar = 20 mm.

One nearly complete centrum displays broken surfaces both anteriorly and posteriorly, and is therefore identified as being from a middle sacral vertebra. This centrum measures about 25 mm long, and is considerably longer anteroposteriorly than deep dorsoventrally. The anterior end is about as transversely wide as dorsoventrally deep, but the posterior end is considerably wider transversely than deep dorsoventrally. A large articular facet for the sacral rib is present on the anterodorsal corner of the lateral surface of the centrum. There is also a slight depression, which may represent a shallow pneumatic fossa, near the midlength of the lateral surface (Fig. 3a). The ventral surface of the centrum is rounded.

A nearly complete caudal vertebra is probably one of the anteriormost members of the caudal series. The centrum’s anteroposterior length is about 15 mm, much less than its dorsoventral height (about 24 mm). Both the anterior and posterior articular surfaces of the centrum are concave, and their edges are strongly everted laterally so that the midlength portion of the centrum is constricted (Fig. 3h,i). The lateral and ventral surfaces are relatively flat, although the ventral surface is narrow. The transverse process projects laterally and is relatively slender in dorsal view, tapering further near the distal end (Fig. 3f), a feature also seen in *Falcarius* but absent in most other therizinosaurs including *Jianchangosaurus*, *Suzhousaurus*, and *Neimongosaurus*^4,18,19^. The dorsal surface of the transverse process is flat, and directed somewhat posteriorly due to distortion, whereas the ventral surface is convex because of the presence of a rounded ridge formed by the confluent anterior and posterior centrodiapophyseal laminae (Fig. 3i). The prezygapophysis extends anterodorsally, with the facet facing mediodorsally.

**Figure 4:**
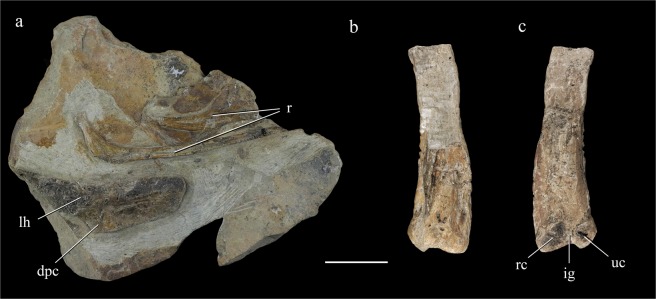
Humeri and ribs. Left humerus and ribs (**a**); right humerus in posterior (**b**) and anterior view (**c**). Abbreviations: dpc, deltopectoral crest; ig, intercondylar groove; lh, left humerus; r, ribs; rc, radial condyle; uc, ulnar condyle. Scale bar = 30 mm.

Several dorsal ribs are present, but they are incompletely preserved and exposed on only one side. Most of the dorsal ribs are middle and posterior ones, as indicated by their extremely short tubercula. The best-preserved dorsal rib is slender, and its nearly straight shaft has a preserved length of about 120 mm. The tuberculum is broken but can be inferred to have been extremely short. The capitulum is slightly expanded and is connected to the shaft by an elongated neck, which forms an obtuse angle of 122° with the shaft (Fig. 4a). A narrow groove extends along the posteromedial edge of the shaft.

The damaged proximal portion of the left humerus and the nearly complete distal half of the right humerus are both preserved. The left humerus is exposed in anterior view (Fig. 4a), displaying an elongated, rectangular deltopectoral crest that curves anteriorly as in *Alxasaurus*^3^. The humeral head projects proximally and seems to be somewhat ball-like. The distal half of the humerus is curved in the medial direction. Unlike in most other therizinosaurs, the humeral distal end lacks any significant transverse expansion. The radial and ulnar condyles are both situated on the anterior surface of the humeral shaft, the former exceeding the latter in size. An intercondylar groove is present and runs onto the distal articular surface of the humerus.

**Figure 5:**
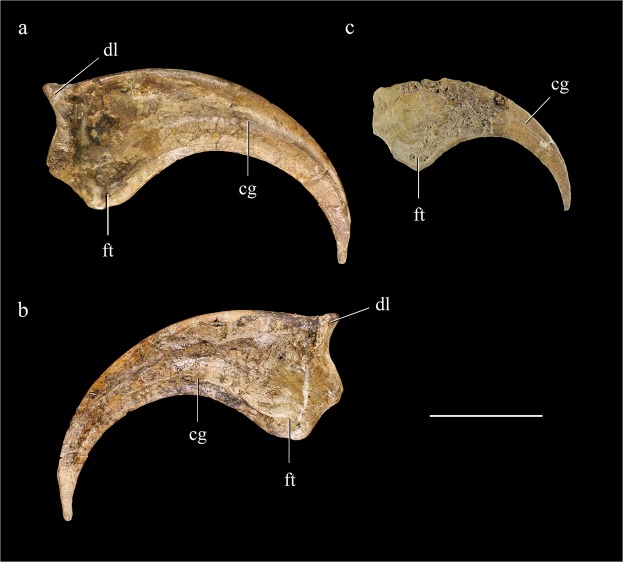
Manual unguals. Probable manual ungual III-3 in lateral (**a**) and medial view (**b**); probable manual ungual IV-4 in lateral view (**c**). Abbreviations: cg, collateral groove; dl, dorsal lip; ft, flexor tubercle. Scale bar = 30 mm.

There are two preserved manual unguals, one of which is only 70% the length of the other. The larger one is probably a manual ungual III-3, while the smaller one is probably a manual ungual IV-4. The ungual III-3 is nearly complete and free of matrix, but IV-4 is exposed only on one side, which is damaged over most of its surface. Both unguals are gracile and transversely compressed. They are strongly recurved as in most therizinosaurs other than *Therizinosaurus*, in which the unguals are relatively straight^2^. They both possess large, proximodistally elongate flexor tubercles, which are located slightly distal to the proximal end. Proximally, the collateral grooves become shallow and wide, and they are continuous with large depressions on the proximal portions of the medial and lateral surfaces of the unguals (Fig. 5a,b). A similar condition is seen in *Jianchangosaurus*, but proximal depressions are absent in *Falcarius* and *Beipiaosaurus*^4,18^. Distally the collateral grooves are narrow, with sharp ventral margins. As they extend distally, both the medial and lateral collateral grooves pass onto the dorsal surface of the ungual well proximal to the ungual tip. This may represent a diagnostic feature for the Therizinosauroidea, as collateral grooves that extend onto the dorsal edge of the distalmost part of the ungual seem to also be present in *Beipiaosaurus*, *Alxasaurus* and *Therizinosaurus*. The distal portion of the lateral surface of both III-3 and IV-4 is flat. The non-grooved ungual distal portion appears to be triangular in cross section, with flat medial, lateral, and dorsal surfaces at least in III-3. This type of transversely broad ungual tip is also seen in manual phalanx III-3 of *Beipiaosaurus* IVPP V11559. A moderately strong proximal’lip’ overhangs the proximal articular surface of III-3 as in *Falcarius*, *Alxasaurus* and *Erliansaurus*^3,20,21^.

**Figure 6:**
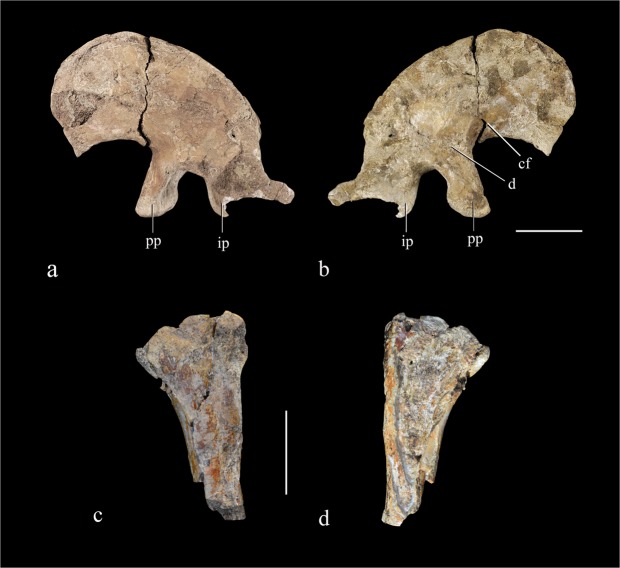
Left ilium and left ischium. Left ilium in lateral (**a**) and medial view (**b**); left ischium in lateral (**c**) and medial view (**d**). Abbreviations: cf, cuppedicus fossa; (**d**), depression; ip, ischial peduncle; pp, pubic peduncle. Scale bar = 30 mm.

The left ilium is nearly complete, despite some damage to the posteriormost part of the bone. It measures 143 mm in maximum length and 95 mm in height at the level of the pubic peduncle. The preacetabular process is more than 1.6 times as long anteroposteriorly as the postacetabular process. The dorsal edge of the ilium slopes anterodorsally so that the long axis of the iliac blade forms an angle of about 38° with the horizontal when the pubic and ischial peduncles are placed at the same level, a feature also seen in most other therizinosaurs^19,22–24^. The anterior margin of the preacetabular process is strongly convex in lateral view, but the convexity ends abruptly at a pointed ventral process which lies significantly posterior to the anterior extremity of the ilium. The ventral portion of the preacetabular process flares laterally, but the flaring is far less pronounced than in many therizinosaurs including *Falcarius*, *Neimongosaurus*, *Nothronychus*, *Suzhousaurus*, *Nanshiungosaurus*, and *Segnosaurus*, in all of which the ventral edge of the process displays a strong lateral deflection^18,19,23–26^. The lateral surface of the ilium lacks a vertical ridge situated over the pubic peduncle, as in some therizinosaurs such as *Segnosaurus* and *Nanshiungosaurus*^23,24^. However, the dorsal margin of the ilium bears a lateral flange slightly anterior to the level of the pubic peduncle, and this flange might be homologous to the vertical ridge seen in other therizinosaurs. Most of the dorsal margin of the ilium is strongly convex in lateral view, but the dorsal margin of the postacetabular process is concave, a feature unknown in other therizinosaurs (Fig. 6a,b). The pubic peduncle is elongated dorsoventrally, although not to the degree seen in some late-branching therizinosaurs. The peduncle is slightly constricted at mid-length owing to the concavity of the posterior margin, as in *Beipiaosaurus*, *Suzhousaurus* and *Nanshiungosaurus*^19,22,24^, but its anterior margin is nearly straight in lateral view rather than convex as in many other therizinosaurs^19,23^. The distal articular surface of the pubic peduncle faces posteroventrally, a feature seen in many other therizinosaurs including *Segnosaurus*, *Nanshiungosaurus*, and *Suzhousaurus*. In *Falcarius* and *Beipiaosaurus*, however, the corresponding articular surface is directed ventrally, as is typical of theropods^18,19,22–24^. As in *Beipiaosaurus* IVPP V11559, the anterior half of the pubic peduncle is laterally depressed to contribute to the cuppedicus fossa. The ischial peduncle is partially broken away, but can be inferred to have had a relatively blunt distal end as in other therizinosaurs^18,19,22–24^. There appears to be an acetabular wall along the posterior margin of the pubic peduncle, resulting in partial closure of the acetabulum, but this region is poorly preserved. The supraacetabular crest seems to be prominent, though this region is damaged. The medial surface of the iliac blade is slightly convex dorsoventrally. At the base of the pubic peduncle the medial surface bears a distinct triangular fossa, whose anterior border is defined by a short ridge that also forms the dorsal border of the cuppedicus fossa.

A fragmentary bone is tentatively identified as the damaged proximal portion of the left ischium (Fig. 6c,d). Although both the pubic and iliac peduncles are incomplete, they are inferred to have respectively been relatively small and relatively large, reflecting a combination of plesiomorphic and apomorphic morphology.

**Figure 7:**
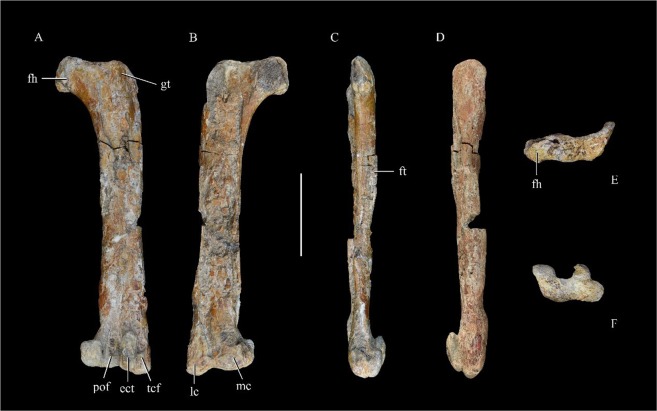
Right femur in posterior (**a**), anterior (**b**), medial (**c**), lateral (**d**), proximal (**e**) and distal view (**f**). Abbreviations: ect, ectocondylar tuber; fh, femoral head; ft, fourth trochanter; gt, greater trochanter; lc, lateral condyle; mc, medial condyle; mm, medial malleolus; pof, popliteal fossa; tcf, trochlea fibularis. Scale bar = 50 mm.

The right femur is nearly complete, but its anterior surface is severely damaged. The length of the femur is 200 mm. The femur is proportionally robust as in most therizinosaurs^4,19,26^, but in contrast to the usual condition in non-therizinosaurian theropods, the ratio of length to mid-shaft diameter being about 7.0. This ratio ranges from about 5.0–9.0 in most therizinosaurs, but is about 11.0 in, for example, *Velociraptor* IGM 100/986. The femur is nearly straight in lateral view as in the therizinosaurs *Jianchangosaurus*, *Beipiaosaurus*, *Erliansaurus* and *Neimongosaurus*^4,5,20,25^, but the femur is sigmoid in *Segnosaurus* and anteriorly bowed in *Falcarius*, *Suzhousaurus* and *Alxasaurus*^3,19,23,27^. The femoral head is dorsomedially oriented with a distinct neck. This feature is also seen in some other therizinosaurs, including *Alxasasurus*, *Erlianosaurus*, *Suzhousaurus* and *Nothronychus*, whereas in *Falcarius*, *Jianchangosaurus*, and *Beipiaosaurus* the femoral head is oriented straight medially^4,5,18,19,26,28,29^. The posterior surface of the femoral head bears a wide ligament sulcus that extends dorsomedially. The greater trochanter is anteroposteriorly much wider than the femoral head, and proximally separated from the latter by a wide, shallow groove. This groove is deeper in *Alxasaurus*, *Neimongosaurus*, *Erliansaurus*, *Suzhousaurus* and *Nothronychus*^3,19,20,25,26^, but is absent in *Falcarius* and *Jianchangosaurus*^4,18^. The anterior trochanter is broken, but appears to be fused with the greater trochanter to form a trochanteric crest, a feature also seen late-branching therizinosaurs such as *Erlianosaurus*^20^. About 20 mm distal to the trochanteric crest is a low trochanteric shelf, anterior to which is a longitudinal groove on the femoral lateral surface. This groove defines the anterior border of the anterior trochanter, the lateral surface of which is somewhat rugose. A fourth trochanter is present along the posteromedial margin in the form of a low rugose ridge about 35 mm long. The fourth trochanter arises proximally at about the level of the distal end of the trochanteric shelf, and extends distally to a level near the mid-length of the femoral shaft. The proximal and distal ends of the trochanter both recede smoothly into the shaft’s main surface. A long, distally extensive fourth trochanter is evident in most therizinosaurs, but is absent in *Jianchangosaurus* and *Erliansaurus*^4,20^. The distal end of the femur bears two prominent condyles, separated by a broad and deep groove that extends onto the distal articular surface of the femur. The lateral condyle extends considerably farther distally than the medial one. A proximodistally long ectocondylar tuber (tibiofibular crest) is present (Fig. 7a). The ectocondylar tuber is mediolaterally narrower than the medial condyle, but extends farther proximally than the latter. A proximodistally long ectocondylar tuber is present in all known therizinosaurs^18–20,25,26^. The medial condyle and ectocondylar tuber are approximately equal in posterior prominence, and the distal surface of the ectocondylar tuber and the posterior part of the distal surface of the medial condyle are separated by a distinct step from the main part of the distal articular surface of the femur. A notch is even visible in lateral view between the ectocondylar tuber and the main part of the distal articular surface, though this feature has probably been exaggerated by poor preservation. Lateral to the ectocondylar tuber is a broad subdued area on the posterior surface of the lateral condyle (Fig. 7a), as in *Falcarius* and *Suzhousaurus*. The popliteal fossa between the two condyles is distally open.

**Figure 8:**
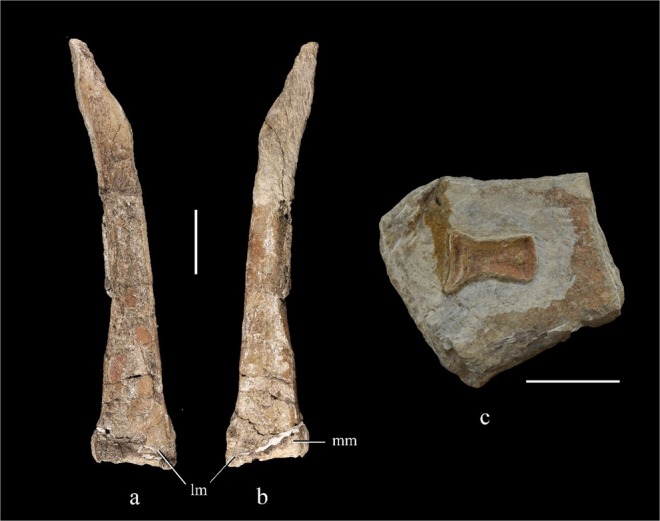
Left tibia and astragalus. Left tibia in anterior (**a**) and posterior view (**b**); right astragalus in proximal view (**c**). Abbreviations: lm, lateral malleolus; mm, medial malleolus. Scale bar = 30 mm.

The tibia is represented by the poorly preserved left element, which is missing its proximal end. The preserved portion of the left tibia measures about 215 mm, indicating that the tibia was longer than the femur. This feature is shared with *Falcarius* and *Jianchangosaurus*^4,27^, whereas in more late-branching therizinosaurs such as *Segnosaurus*, *Neimongosaurus*, *Erliansaurus* and *Nothronychus*^20,23,25,26^ the femur is longer than the tibia. In anterior or posterior view, the tibia widens by a factor of about 1.8 from the mid-shaft area to the distal end (Fig. 8a,b). The distal end of the tibia is anteroposteriorly compressed and mediolaterally expanded. In anterior view, the lateral edge of the lateral malleolus is straight, while the medial edge of the medial malleolus is bluntly rounded. The lateral malleolus extends farther distally than the medial one as in *Neimongosaurus*^25^, so that the distal articular surface of the tibia is canted medially. In *Nothronychus*, by contrast, the malleoli are equally extensive distally^25,26^. The distal margin of the tibia appears slightly concave in anterior and posterior view.

We tentatively identify an isolated element that remains embedded in matrix as a right astragalus. The main body is broken away, with only the bone forming the distal surface of the astragalus being exposed in proximal view. The proximal surface of this sheet of bone is concave anteroposteriorly and slightly convex mediolaterally (Fig. 8c). The lateral portion has a semilunate outline, fitting the shape of the calcaneum.

## Methods

Phylogenetic analysis. To investigate the systematic position of *Lingyuanosaurus sihedangensis*, we conducted a numerical phylogenetic analysis on a modified version of a dataset compiled by Sues and Averianov (2016) to elucidate the relationships of non-avian coelurosaurian theropods^30^. We revised seven characters in the original dataset and added four new characters (see Appendix). Forty-five characters were scored for *Lingyuanosaurus*, representing about 12.7% of the total characters in the matrix. Twenty-one characters (characters 27, 37, 40, 68, 76, 78, 97, 106, 113, 157, 163, 168, 253, 303, 304, 308, 309, 310, 334, 342, and 345) were designated additive and two characters (characters 165 and 215) were excluded. The dataset was analyzed using TNT version 1.1 with equally weighted parsimony and traditional search methods on 1000 replicates of Wagner trees with random addition sequences, and subjected to tree bisection-reconnection (TBR) swapping methods holding 10 trees per replicate, followed by a second round of TBR in order to ensure detection of all possible most parsimonious trees^31^.

**Figure 9:**
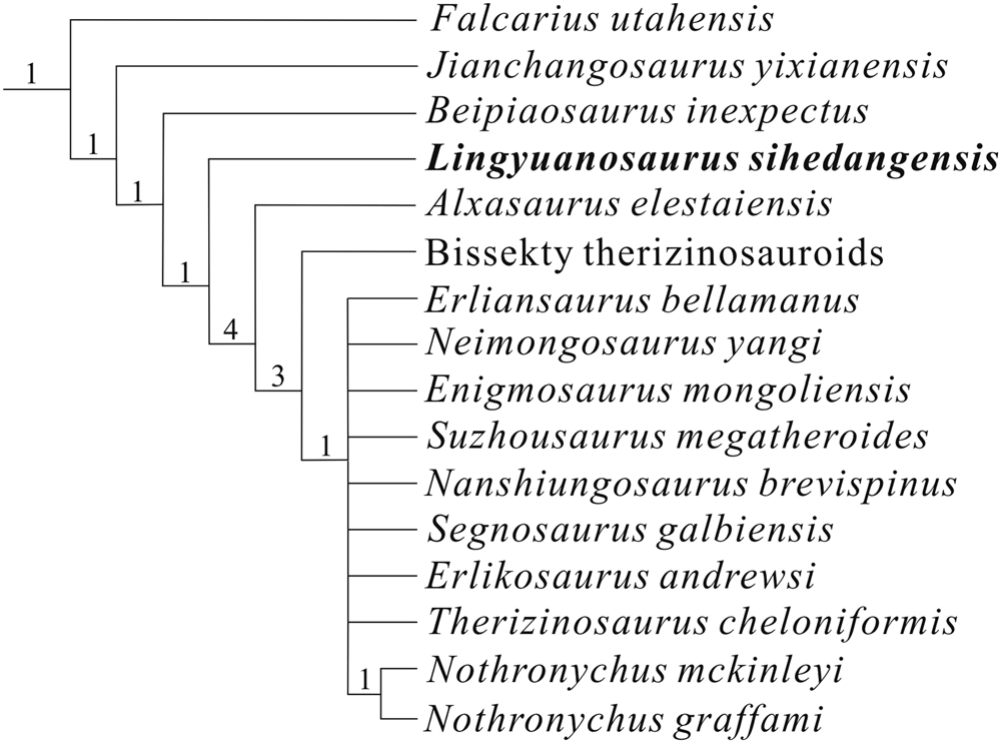
Strict consensus of 27648 most parsimonious trees. The numbers above the nodes represent Bremer support values.

The phylogenetic analysis resulted in 27648 most parsimonious trees, each with a length of 1311 steps, a consistency index of 0.344 and a retention index of 0.694. The strict consensus of the most parsimonious trees placed *Lingyuanosaurus* within Therizinosauroidea, in a position between early-branching therizinosaurs such as *Falcarius*, *Jianchangosaurus* and *Beipiaosaurus* and late-branching therizinosaurs such as *Alxasaurus* and other species (Fig. 9). We also calculated Bremer support values to assess the robustness of the recovered clades, which indicated that most clades were poorly supported (with a Bremer support value of only 1). To test the robustness of our phylogenetic results, we analyzed a modified version of our matrix from which most non-therizinosaurian taxa and numerous uninformative characters were removed, but obtained the same strict consensus topology (see Supplementary Information).

## Discussion

Despite the incompleteness of this specimen, IVPP V 23589 is clearly a therizinosaurian dinosaur as indicated by numerous therizinosaurian synapomorphies^2^: dorsal vertebrae with a complex laminar structure; laterally flattened manual unguals with dorsally positioned collateral grooves; and a highly modified ilium with a deep preacetabular process, a reduced postacetabular process, a preacetabular process whose ventral margin is dorsally displaced relative to the acetabulum, and a dorsoventrally elongated pubic peduncle. These features are present in all known therizinosaurs^2^, contributing to the bizarre therizinosaurian body plan.

**Figure 10:**
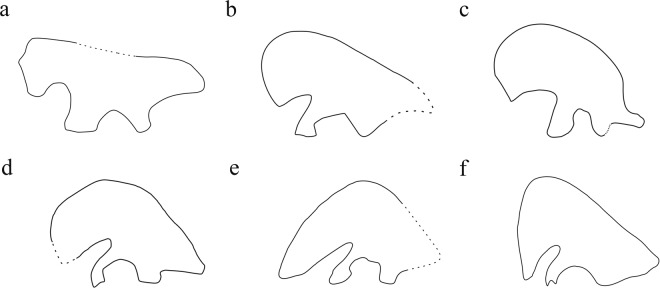
Schematic drawings of therizinosauroid left ilia in lateral view; dashed lines represent reconstructed parts of the outlines of the ilia. (**a**), *Falcarius utahensis*, modified from Zanno (2010a); (**b**), *Beipiaosaurus inexpectus*; (**c**), *Lingyuanosaurus sihedangensis*; (**d**), *Suzhousaurus megatherioides*, modified from Li et al. (2008); (**e**), *Nanshiungosaurus brevispinus*, modified from Dong (1979); (**f**), *Segnosaurus galbiensis*, modified from Barsbold and Perle (1980). Not to scale.

*Lingyuanosaurus* is similar to late-branching therizinosaurs in having the following apomorphic features: groove ascending proximal to entepicondyle on anterolateral margin of humeral shaft absent; ventral margin of preacetabular process of ilium gently deflected laterally; articular surface of pubic peduncle caudoventrally directed; long axis of iliac blade inclined above horizontal at an angle exceeding 35° (Fig. 10); femoral head dorsomedially oriented; greater trochanter significantly expanded in craniocaudal width relative to femoral head; proximal surface of femur depressed between greater trochanter and femoral head. However, *Lingyuanosaurus* also resembles early-branching therizinosaurs in possessing many plesiomorphic features: tibia longer than femur; lateral femoral distal condyle extends farther distally than medial condyle; transverse processes of anterior caudal vertebrae taper distally; lack of pleurocoels on lateral surfaces of anterior caudal vertebrae; dorsal margin of postacetabular portion of ilium smooth, rather than rugose; preacetabular portion of ilium subequal in height with portion directly dorsal to center of acetabulum; and manual ungual III-3 with dorsal ‘lip’ overhanging proximal articular surface.

As indicated by the combination of plesiomorphic and apomorphic features present in *Lingyuanosaurus*, and confirmed by the formal phylogenetic analysis, this taxon represents a therizinosaur intermediate in grade between early-branching and late-branching members of the group. As such, *Lingyuanosaurus* provides significant new information on the evolution of major therizinosaurian characteristics. The highly modified pelvic girdle and appendage of therizinosaurians are among the most bizarre structures known in any theropod dinosaur, and have been interpreted as adaptations for a lifestyle characterized by herbivory and, relative to other theropods, reduced cursoriality^2^. Some previously described early-branching therizinosaurs, such as *Falcarius* and *Beipiaosaurus*, have shed considerable light on how the specialized pelvic girdle and appendage morphology seen in late-branching therizinosaurs evolved from the typical theropod condition^5,27^. *Lingyuanosaurus* provides additional information with respect to patterns of anatomical change during this transition.

The therizinosaurian ilium displays an evolutionary trend involving deepening of the preacetabular process, stronger lateral flaring of the ventral portion of the preacetabular process, reduction in the size of the postacetabular process, increased anterodorsal inclination of the iliac dorsal margin, dorsoventral lengthening and anteroposterior narrowing of the pubic peduncle, and deflection of the distal articular surface of the pubic peduncle to face more posteriorly (Fig. 10). With regard to these morphological changes, *Lingyuanosaurus* shares with late-branching therizinosaurs an extremely deep preacetabular process, a highly reduced postacetabular process, a strongly inclined iliac dorsal margin, and a posteroventrally facing distal articular surface of the pubic peduncle^19,23,24^, and shares with *Beipiaosaurus* an intermediate degree of narrowing of the pubic peduncle. Specifically, the peduncle is considerably longer dorsoventrally than wide anteroposteriorly in *Lingyuanosaurus* and *Beipiaosaurus*, being intermediate in its proportions between the more robust pubic peduncle seen in *Falcarius* and the even more slender one seen in late-branching therizinosaurs. The preacetabular process is plesiomorphic in that the ventral edge of the preacetabular process flares slightly laterally, a condition shared with *Beipiaosaurus*, whereas the flaring is somewhat more pronounced in *Falcarius* and much more pronounced in late-branching therizinosaurs^18–20,23–26,32^. Information from *Lingyuanosaurus* thus demonstrates that the evolution of the therizinosaurian ilium was not a simple linear process and that different parts of the ilium were modified at different rates, resulting in a mosaic distribution of iliac character states in various taxa. Narrowing of the pubic peduncle appears to have taken place in a progressive, gradual fashion in therizinosaurian evolution, with the peduncle being wide in the early-branching *Falcarius*, intermediate in its proportions in the transitional taxa *Beipiaosaurus* and *Lingyuanosaurus*, and narrow in late-branching therizinosaurs. Lateral flaring of the ventral margin of the preacetabular process, by contrast, is better developed in *Falcarius* than in *Beipiaosaurus* and *Lingyuanosaurus*, suggesting either that flaring was independendently accentuated in *Falcarius* and late-branching therizinosaurs or that the degree of flaring was reduced in the early stages of therizinosaurian evolution before secondarily increasing to an unprecedented degree in late-branching taxa.

The evolution of the therizinosaurian hindlimb displays a similar pattern. In general, the therizinosaurian hindlimb shows an evolutionary trend towards reduced cursoriality, as indicated by increased robustness of the hindlimb bones and proportional shortening of the lower segments of the hindlimb. *Lingyuanosaurus* shares with *Falcarius* and *Jianchangosaurus* a tibia that is longer than the femur^4,18^, a plesiomorphic condition widely present in theropods, whereas in most other therizinosaurs the femur is longer than the tibia^20,25,26^. However, *Lingyuanosaurus* shares with late-branching therizinosaurs a straight and robust femur with a reduced anterior trochanter, in contrast to the anteriorly bowed, less robust femur with a large lesser trochanter seen in *Falcarius* and several other basal therizinosaurs^4,5,18^.

The discovery of *Lingyuanosaurus* adds to the known dinosaurian diversity of the Jehol Biota. Two therizinosaurs, *Jianchangosaurus* and *Beipiaosaurus*, were previously recovered from the Yixian Formation of Liaoning Province, which forms part of the Lower Cretaceous Jehol Group. *Lingyuanosaurus* thus represents the third therizinosaur from the Jehol Group^4,5^, irrespective of whether the strata in which it occurs are ultimately referred to the Yixian Formation or the overlying Jiufotang Formation. Furthermore, these three species are similar in body size. The presence of three similar-sized therizinosaurs in the Jehol Group is unusual, given that competitive exclusion might be expected to prevent this situation, but has several possible explanations. First, the beds in which these therizinosaurian dinosaurs occur are not precisely dated, and the Jiufotang and Yixian formations of the Jehol Group were deposited over a span of at least eight million years^33^. Accordingly, it is possible that *Jianchangosaurus*, *Beipiaosaurus* and *Lingyuanosaurus* were separated from one another by substantial spans of geological time. Second, these three species are known from different localities, although the intervening distances are small. Some limited evidence suggests that deposition of the Jehol Group occurred in multiple small basins, suggesting that the three Jehol therizinosaurs might have been separated by geographic barriers even if they were mutually contemporaneous. Finally, it is possible that *Jianchangosaurus*, *Beipiaosaurus* and *Lingyuanosaurus* all occupied disparate ecological niches. The middle and posterior dentary teeth of *Jianchangosaurus* are morphologically distinctive, presumably indicating an unusual type of feeding behavior^4^, which could have resulted in resource partitioning between *Jianchangosaurus* and the other two Jehol therizinosaurs. Similarly, the shortness of the femur relative to the tibia in *Beipiaosaurus* implies that this taxon was less cursorial than *Lingyuanosaurus* and *Jianchangosaurus*, suggesting an ecological difference rooted in locomotion.

## Supplementary information

Supplementary Information

## Data Availability

All data generated or analyzed during this study are included in this published article (and its Supplementary Information Files).
