# Supplementary material for: A new transitional therizinosaurian theropod from the Early Cretaceous Jehol Biota of China
Source: Sci Rep. 2019 Mar 22;9:5026. doi: 10.1038/s41598-019-41560-z (PMC6430829; doi:10.1038/s41598-019-41560-z)
Supplement: Supplementary file 1 — Supplementary Information [file 41598_2019_41560_MOESM1_ESM.pdf]

A new transitional therizinosaurian theropod from the Early Cretaceous Jehol Biota of China

Xi Yao, Chun-Chi Liao, Corwin Sullivan, Xing Xu

1. Phylogenetic analysis

In an attempt to determine the phylogenetic position of *Lingyuanosaurus* with greater precision, we reanalyzed our matrix following removal of all non-therizinosaurians other than *Allosaurus*, which was retained as an outgroup taxon. In this modified matrix we also deleted 143 invariable characters, leaving 17 taxa and 214 variable characters. The reduced dataset was analyzed using TNT version 1.1 with equally weighted parsimony and traditional search methods on 1000 replicates of Wagner trees with random addition sequences, and subjected to tree bisection-reconnection (TBR) swapping methods holding 10 trees per replicate, followed by a second round of TBR in order to ensure detection of all possible most parsimonious trees.

The phylogenetic analysis resulted in 32 most parsimonious trees, each with a length of 295 steps, a consistency index of 0.842 and a retention index of 0.772. The strict consensus recovered the same topology as in our first analysis (Fig.1). We also calculated Bremer support values, which indicated relatively strong support for all but three nodes (which had Bremer values of only 1).

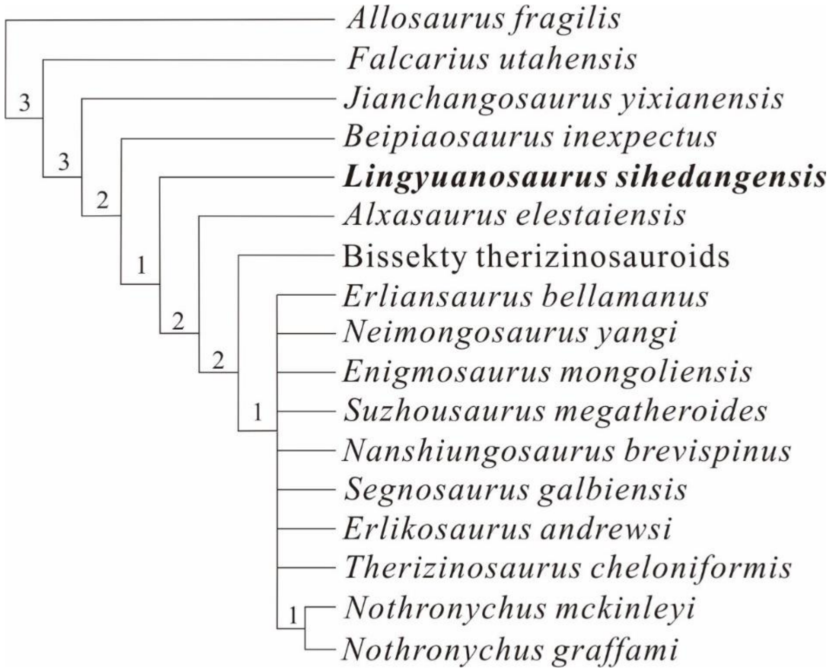

Figure 1. Strict consensus of 32 most parsimonious trees obtained from analysis of a modified version of the data matrix used in this study. The numbers above the nodes represent Bremer support values.

2. Newly added characters

349. Dorsal surface of distal portion of manual ungual III-3
    0: rounded
    1: flattened
350. Femur in lateral view
    0: curved
    1: nearly straight
351. Lateral distal condyle of the femur
    0: extends farther distally than the medial condyle
    1: extends about as far distally as the medial condyle
352. Anterior caudal vertebrae, appearance of transverse processes in dorsal view

0: tapered distally

1: rectangular or slightly expanded distally

3. Modified characters

105. Zygapophyses of trunk vertebrae

0: abutting one another above neural canal, opposite hyposphenes meet to form lamina

1: placed lateral to neural canal and separated by groove for interspinous ligaments, hyposphenes separated

2: abutting one another above neural canal, opposite hyposphenes meet ventrally and form a transversely expanded intumescence.

155. Preacetabular part of ilium

0: roughly as long as postacetabular part of ilium

1: more than 1.5 times length of postacetabular part of ilium

158. Postacetabular ala of ilium in lateral view

0: squared

1: acuminate

2: reduced, iliac blade terminates at rectangular end just posterior to level of acetabulum

209. Neural spines on caudal dorsal vertebrae in lateral view

0: rectangular or square

1: fan-shaped, with craniocaudally expanded dorsal ends

2: dorsal borders curved, but not craniocaudally expanded

304. Ventral edge of blade of preacetabular portion of ilium

0: parasagittal, in line with dorsal portion

1: gently deflected laterally

2: strongly deflected laterally, nearly perpendicular to parasagittal plane of ilium

307. Angle between dorsal margin of ilium and line connecting articular facets for pubis and ischium

0: less than 15 degrees (gently inclined)

1: between 15 and 35 degrees (moderately inclined)

2: more than 35 degrees (steeply inclined)

310: Pubic peduncle of ilium

0: straight

1: anterior margin straight, posterior margin curved, articular surface ventrally directed

2: anterior margin straight, posterior margin curved, articular surface caudoventrally directed

3: anterior and posterior margins both curved, articular surface caudoventrally directed

4. Matrix used in the phylogenetic analysis

Allosaurus fragilis

?11000?000000010001100100010000001110110010??0000000000100010000000000000010
0000000000101011010000000100101000000000000001000000?01????000000000001000000000001
000000100000000100000000100?1100001100011010000000000000000000000100000000000?0?000
?000100001000000000000000000000000000000002010000000000000000000000000000000000000
00?0000000?0000000000?000000000?0000?0??

Sinraptor dongi

?11000?000?00010001000000010?0000010110010?00?000000000000010000?00000000?10?
0000000001010100100000001001010000000000??????????1?00?0?0??00?????????000?10000
?010??00001000000002?001000000?0000101000000000000?000?000?00?000000?0?000?0001
0000111100010?00000000000000000100100000??000???????????00?0??0000000100001?0000
000?0000000?0???0??0000??0000????

Ingenia yanshani

?01?0????????????????1?1?????1??????????????????????????????????21120301000?0111?2
???????????????????????????1???01????2?00???0011110011??00000??1010001000021??????01
01202001101??11101101?0000?00010?000000000??0000??11?2000?000?0?001000????????????
?????10??1?111?????1??1?0?????1111101200001101001011100000000101000?0000111?020?000
000011001?000???00????

Citipati osmolskae

?0110?1001001??221000101111?01011?000102100011000001100010001101211203010001
0111?2?????????10111011001011?1001??201??002100111?0111100110100100?1101000010002
11001??01012020?010111111?2101000000000?0000000000000000000011020000000?0?001000?
000002011???0?2020?110111??????????????????1???100?00??0???1000100?0???????????0????
??????????0001?00???00??100????

Oviraptor mongoliensis

?01?0????0??????????0111?1???1?11?00010??00???000?0??????00??1?12112?301000??111
?2???????????????????????????????0?????210?????????1??1???00?0???101?00??000?1?????1??
?????????????1?????????0????0??0?0???????0??00??001??2?00??????????????????????00???????
????1011??0???????????????????????????????????
?????00????

Oviraptor philoceratops

?01?0??????01?1????0??1??111?1011???0???1?0?11????0?1?0???0?11?121120301?00?01?1
?2??????????????????????????????0????????????????11??1?????00???1?1??0??00??1??0??????
?????????????????????0????0????0??0?????00?00000???2?00?0????????????10000?0?0???????
1??110111??????????????????1?00100??0???1?0100?10??0??????????????????????????????????
???????00????

Conchoraptor gracilis

?0110??????????1???00111?1???1?11?000??21000110?000?????1?0????1211203010?0?011
1?2???????????????????010????012?110??1?????0??1???1?10011000010????????0010002100010
101?0??20???01101?1?11010?0????0??0?0000??0?0??000?001102?0?0000?0?001000000000200
1?????????0110??10001?0?00???0??????????????????????
0?1?0????????1??????

Incisivosaurus gauthieri

?01?01??11?01?????1000?1101001001?010?1?000011000?11010010101111200103001001?
1?001?20??00?0??
??00??00??01?0??0?0?????????000000001?0010
2201?0100110010?1???
??0????1000????

Microvenator celer

??21?2030????????????
?????????011???11000112100???0?1?1002????????????00?000?11000??????00?00021??0000???

???2??????1110??0101100?0000010???????0??0???00??11??0?0??00???0?1??0???????????????
???????101?????????00?001??0????110000?021?0?????0000000010???00001?1?11??00000???
???1?000??????01?

Chirostenotes pergracilis

?????1??11?01101??0???1?110?0????????????????????????01010??????21120100000?01??
?2?????????????????1101?11????1?12???0?????????????????0??1???????????00220?02??00?0?01
2120?01110??11???0?????????00?100?00200000????0?0?1??20?0?000?0?00100??????????????
?0020?1??110?????11120?10??111????????????????001?01000??0??010?1??0000?1?????0???000
1?00??00????????0?

Dromaeosaurus albertensis

?0??0010000000000010??0???0?01??01110????1111????10001?1001100??000000000111110
00?0000101001???1???????
?????????????????????????????????????1??????00??????00?0?00??1?????????1?00??01100010
000??10000000000???0?1???
?0????0??0????

Deinonychus antirrhopus

?0110????1???????1??0000?0111000111000011??????0?1????0?00?110100000?0000111?100
0?0100101000?110001100?1112011????0011011121??????1?11011100100111010000100221111
0101010220?01111?01201?1111000000000010001101001000?000001100000?00010?0010001010
00001111011???0??00000???001000000000?00??004000001?010000100000 0?0000000??0?????
????000?0?0001100?0000?0000????

Velociraptor mongoliensis

?0110010010000120112000010111000111000012111?10?01000100001110100000000001111
10001110010100001100111001111101111100011011012111001110111111001001110100001 0022
1110111010220201112212011111?000000000101011010010000000001100000000011?00100010
1000001110011?000110?000000001000 00?0001000100400000011010?10000000000000010?0?00?
0001?010000??0001100000000?00003????

Utahraptor ostrommaysorum

?????????????????????0???????????????001??0???
?0101??1????????????1????????????0??011????????????????????????????????0??????????????????
??????1??????0??????????00?0????0??
????0??00?????????????0??0?0?????????????0???0?
??????

Adasaurus mongoliensis

???0?0??
0?????????????????????2??1?22111?10?01?0202
?1?1?2210?1????????????????????01???0??????0????0???????1??0?10????????????????????01?
??????????0??1000?00??0?100?????????????????????00000000000?000?001??100000000?11??0?0
0????00????

Achillobator giganticus

????????????????????????01?1??00
00101??????0?01100?11120???????0??011?11??????????101?????????????00??10220??1101000
2101?011011?001?21110?0????00?000?101????0???????1???0????001??00100011100??0??100?
????0??????????000???00???0??0?1?0???????????0?????0000?00001?00?0001?01???00??????01

???000???0???????

Tsaagan mangas

?01100100100001201120000101010001?10001211?110001000??00001?0??000000000?111
10001010010100001100??1??????????????????????????????????11?11?????????????????????????
???0??00??00?0?00???????????1110000011?0
0?0001?11??000000000000??????????1???
?????0????0000????

Saurornitholestes langstoni

??111???????????????11????0?????????????????1
00101?00?11000110011112011011100?1011?111?????????????????????????0002??????1?????1?
0??????????????????????????????????0????0???00?0????00??000010?001000????????1110011?
?????????????00????????????????????????????????????00??????????????????????????????????????
?????0?0????

Sinornithosaurus millenii

0011??????0??????????00???1110????1000111100?1???0?01???????????00000000??1????0
10100??1001???????1??1???????0???00?1??1???????0111?11011110?0????0?0000?00?201?01?1
11023?2?111020?2?1???1?????????110001?1100?0?00010001??0000???01101111001000000111
100?0????????00001000???1?????0??01?0040????????0?1010000?????00?0?01000????????????
?0001?00?0?00?0000????

Microraptor zhaoianus

0??????????????????100???0??000?0?1????0?0
1000000????00?????01?1000?01???0110?12?2111?01?101?1?111010??1001000021012111??01
110?3?2?1110221201?11111??00?0001?10111110?100??000?11?000????101?111100?0?0?????11
0???????????00001?00?????????01?01??0?00??????10??0????0?000?00?0?000000?????00?0?0?
0001?000?000?0???????

Rahonavis ostromi

??
0???????????????01?112???1?01?011112?12????????????0??11??011???????0?01111?10111110?3
01012?01??21??211?0001??0??10001101?0100????0??1????????20001011111?????????????????
?0???????????????????0??01?0?100?????????1?????????00?0000100?00?000???1?0?????00011??
0?00??0???????

Mononykus olecranus

?????100???????112???????????????????????????????????????100?????????????????????????2
????0?????????1?1?11101001?????10?2???????1???1000?000200030210?11?201?00??1????1??00
0?0?2?????2??0???2?0100111011211000030000001??00???1??0?0???0?0?0???00??????????????
???????????????111002100010?0??00022000000000??????00?????00100??00?0001?120001?100
?11001?000?????????

Shuvuuia deserti

?0110?00000000?112011000?0?0?0?111?1100210000101010010010???1?10000100000002
100?11211??000??0111111011101?0??1?201012012110100?1000?00020003011??111201?000?1
??011?2000?0?20021022?0???21010011101121100003000000011000001100000010000?00??000
000000001?0100????11????1???11?????????????????????????????????0?????????????????????????
???????????1????000??0000????

Patagonykus puertai

??
??????????????1?011?11?????12110?2??????????????????00200??0210?1??????????1????1??0????0
20????22101??1?00?0001000?1110??0????0?1??0?0?1???00????????0010?0??????????????????
0???????????????????????0?????2?00??101????????00?????10??0??000??????110?1?10??1100?
?001?????????
Alvarezsaurus calvoi
??
??????????????100???????????2?10?2?12?????????????0?0?00?????????1??????0011??0??2??????
??????????????1?00???0??0??10000000???0????????1???0????00???????0????????????????????
??????????????????????????0???????????????????000000100?0??????0???1???????0??11?????0?
?????????
Ornitholestes hermanni
?0100???010000?1???0?010?01110001?100001?0??100000001?01011????0000001000?0?0
00001?00101001?????011?010110000???000?0010??1???????????????01?00??????????00001??
1?0?010010000001??0?1???????0????????00?00????00000?0010?00?0?0000?0?0000?0000000
??01?0??0?0?01?00011???001001001000???????10000001010??????000000001011?000??????
????0?0?0?1000?0??0?0?00????
Archaeopteryx lithographica
101?0000??000??112010010??1110?011000012100?10?0000?0??100111?0?000000000002?
00001200??00100?1?1???00?0??0?0?0???0021012211000????1011111111000?110100000011211
1010?1100302012?022?212?111100000?0001010000010?00000000001100000??20?00011001000
0001001?0100???0????0000?002???0?????0??011?0300??????1?0101000000?000000?0000000?
?????00???0001?00?0000?0000????
Avimimus portentosus
?01?0???10011?00??0??1?1???????1???1?????00?11??0?00100110??????2?1???00?0??0111
????????????0110101101011?0?00??100?????????????????????0??0100???1?1?????00?211?01??0
101202010?011?0?1000100?0???00011110020???00??0?0?01?02?000000?0?001000????????0??
???????0?????????????2012011101?0??100300000100?1??????????0????1010?0??000011?1000010
10001?001?000??????0??
Caudipteryx zoui
00110???????????????0?111??10?0001?10??121000???0?00??????????????211203000??????0
?2??0??0??01????0??00????????0???01?????20??1??0???01?01??0??0???10100000000?1??????
0121201?1?1????101?11?????0????01000??1?00?0?000000?1102000??00?0?00100000000?00?
???00???0????111?1???1???????????01?100?????????1?01000?00?0?00?01????0?00????????????
?001?00??000?000??0??
Confuciusornis sanctus
10110????????????????1?000?00??0001???0??2??0??0??010???01???????000010000?10
?0001?2???????????0???????????101??0?2???0?2?????2??111?11010??11111400?111110000001
121?1?1??11000?1?111020?21?12??10??0???120111010010?0000000001102000???0?0101??
11?000011001???????????????010????????????????????11????00??????1??101000000?00?000?
0?00000????????0???0001?001?000?0000????
Struthiomimus altus
?0101111111?0??101021000101110 1?110000000000000000001?0??0001?01??0101000001020
001?2?????????001?1011000010000010100000000100100 1?????01120002000000021010010000

0011000010100100000010001000000110001000100000203?000011111110121110000?0?00100000
00000001?????20100000011????????20????0100?000310011202000100000000?0000301???000
000?00000???00011000?000??000????
Gallimimus bullatus
?01011?110110101010210001011001?1100000000000000000100000001?01000100000010
20001?2?????????0011101100001000001?100000000100100??????011200020000000210100100
000011000010100100000011001000000110001000100000203?000001111110121110010?0?001000
000000001????????0????011?????2102??00?0100?000310011?02000100000000000003010?00
00000?00000000000011000?000??000??0?
Garudimimus brevipes
?010?????01101?????2?00010101000??000?00?00?0?000??????0001??1?0?10??0??0?02?0?
1?2???????????????????????????1??0??0?0???1???0?
0?02?????01?0?1???????0?????0??00000000?0??01??1?1??2?1?0?10?0?0??0001000000000????
?20?00??????????????200100010???????????????????????000000010?1?0000000?0000000100
011000?00???000?00?
Pelecanimimus polydon
?01???????1????????2?00????100?????????0??0??????????????????????00?0000???????001
211??0001???????????????????????????????????0?00????2?????00??00?10100???????????????
???????????????????????????????????????001110????1????????????????000?0??0?1?01??????
?????101101??????????????????????11??????1000000?????????????????????????????????????0??
0?00?0????
Harpymimus okladnikovi
?0??????????????????2?0???1??00?00????????22
20??1????????0????????1???0?????????????????????????????2?00?00010010010??0???????010?
0???00????0??????????????????000000???0?001011????1?0???10?0?0???000??0000??1?0??????
0????011????????200?0?0100?0003000112021?0100000000000000301???0???00?0???????00?11
000??00??000????
Troodon formosus
???1?2112?1101000001???0?011?0??????20220000210?00?1?01100????0?10??021????????
???111010100???11?1100101101111?1000?1020??11?????????????1?010???1010000?0????????
?0?3?2?20?11001?0??01111000???00010000021??01????000??1?0??010???1??0??0?0?00000?0?
0?????0???0??000?0??0?????????????0?????????????10??????0?????????????????????????????00
?1100???0?1??00????
Saurornithoides mongoliensis
?01????1??1101???0?110001?1000????????2???????????????????1?010?100?0?10??1????0
011110101?????????1???0??0?????100??1???01
012020?010??10?1?11110??1????00??0???10?01??????0??1?0???????????010??00000??0?1?01?
0???0????00??0000???0001???0?????0001?
0001?0??00?0????
Saurornithoides junior
?01102?12?110100?001?000??100000????2022000?21??0????11100??????100?021???1???
?0011110101000??????????????????1?1000?1020?1??
?1?????????????????????????????01?????2???????????00??00???11????????0??00000000010000
0001?10??00000000??????????1?0???0???1

???1???100?0????

Byronosaurus jaffei

?????2012??101?110011000101 1?0??????20220??????????????100??????000001??11????0
01211??01?0?0???????010110????????0??02???
?????????????????1????0?0??0???????21??????????0????0????1???????????00000000?1?00??00
1??1??000010002??2????????0??00????????????1
???000??????

Sinornithoides youngi

?0??0??????????????1?000??1??0????00???2???????1?0??????????????00??0010???????00
11110?01??????11??001???????????0011?10112101????1?00?1?101?0???1010000????21??01??0
121202??11?01?001??1110?????0?????000110001?0??0?0?1??00?0???0???0010?0??00000????0
1?0???0????00000000?00?????????0?1?0?1???1??21??10?0000????????0?0?00?0?????2000?0?0
00110001000?0???????

Sinovenator changii

?0???2002?0000111100101 01?1110?011?0???2??0011???011?10000??????000?021??????00
00111110?1?????11?10100011010?00100011022 1?1?????????110111???????1010?0?????211?01
011120302?11102?1201?11110000??11010000111??110?00?000???000011?001?001100000000??
1??011??001001??000?1000?001000?001???1?0??0??1???????????0??????000?00?0000?01?0?0
00?0?00011??0100?000?0????

Mei long

?0??????????????????11010?????0?11?000012100001?10?110???00??????00010010??1?000
0?021???01??011011100011?1010?01???0110221111?11????111?11100?0?1?10100002??12111?
1??1100302?1110221???0111100?0101101?00??110011?0?0000???00000???001?0?1??0?0?0000
00?001?0???0????000??0001?0000?00010011?01100??0?01??10?0000????00?0????0?00?????0
0?00?00011000?000?00?0????

EK troodontid

??????012?????????0????????????????????????????????1??0???????????????21??11????????
??1??????0??10100000??????????????????
??????????????????????????????01?1?001???00??????0?0??1?????????0??????????????????1??1
????????00???????????????????????????001010?00??????????????????????????????0?1?????????
????????

Segnosaurus galbinensis

??211?11?0000?000???0
100?001????0???1??????????111?0???????????????????0?10???00100?????0???10011100102010
222??0110232101?11000??0100?31000000021?0??00?01????3?0???00?0?0?0?00?????????0???
????1?????11??111???2??????1?1?0??2010012000???????1?21122232200111111111101011?111
20?111?120??????1??

Erlikosaurus andrewsi

?0110???2?0?1?0??1010011100??0001?1000000000001000?1??00100??11112100110000020
001?10100?001???0????????????????????????
????????????????????????????????0000?021?0??00??11??03?0?02????????????0?0000101001
0?012?01110111?111??????????????????201?001??1?0
?11?0??1?0000????

Alxasaurus elesitaiensis

??210?110????????????
1001001??????????0?01100000?1?0001102?0?????????????0?000??0?100100001?0?11?????1?1
0?????????????????????0?????????00000??0020??001?1??00?0??00?0?????00??????????0?????
???????0110?111????11??10?01???0?0??????01100?01?00????1???1???????111???10???1?1?0?0
01???0??????0??

Tyrannosaurus rex

?10000?01100001002100000101010000000011000010211000010010001?000000000000110
010000100010101101000000?000010200000010000?001000??1?????0000000010100??0??100?10
010001000000010110110010001?010010000?00010000020000000100000000000000010?0?0010
0001000000011000?0100010000000000000000000020100000030001120200?00???100000000001
0?0001100?0100000?000100010000?0111??01

Gorgosaurus libratus

?10000?00?0???????100000101?1000000001100000??21100?010010001????000000000?1001
000010001010110100000??0001020000000100000000100000?10001000000000101000000201000100
1000100000000100100100100010010000000100010000002000000010000000000000?010?0?00100
0??????????????2000000?0000000000000?0?01?000001000?0??00?00000?00000000000010?0
0011????100000?000100010000?0111????

Shenzhousaurus orientalis

???0?????????????????210001?10?0??1???000??00?000?00????????????0?01000000??0??0?1
?2220??1??0?????????0????0???0???00000???0??????????????????????????0000000001??110?0
00010??000002100??00001?0??????????????????0??1??1?0011??????0???0010??000000010?????
?????0??0?0?01?012??????0??0??0????????????????1??000000000000301???00??????0?????????
????0?????00?0????

Ornithomimus edmonticus

?0101??110?101?1010210001010101011000000000000000000010000000??????01000000010?
1001?2???????0?001?101100001000001010000000001001001?????011200020000000210300100
0000110000101001000000110010000001100010001000000203?00?011111110121110000?0?001000
1000000001????????0????011?????2??20?1??010??000310011?020001000000000000?0??????00
0000?0000000?0001?000?000??000????

Archaeornithomimus asiaticus

???
????????00?101100001000001?100000000?0??????????01?200?20000?00?01100??000?1100001
01002?00?001001000001?00010001000000?0?00001?11??10????1??01?00?001000????????????
??????0?????????????2102?0?0?0?00???03100112021001000000??????????0?000???????0000??0
0?1?000?000??????1??

Anserimimus planinychus

???
???????????????????0?????????????????0??????????1???0?2????00021030010000000110000101
00????00??001?????????????????00002?3?00????11??10??1?1??00?0?001000?????????????????
0??????????????????????????00?00011??2??01000000??????????????????????????????01??0??
??0?????????

Huaxiagnathus orientalis

?01?0??????????????0001???1???0?????00??0?????????????????????????00?0000????????00
110?1010?00??0???1????????0??????00???0?100010????000000000?00??0020000000020?0??00

0000100?000012?0???0??????0??0?010000000?0?011??000???00?0??00?0?0010?0000000???1
?00?????0?????00???00???0?????0?00000030??????????00100?0?0000000???????0??????0?????
0001?00?0??0?0???????

Sinosauropteryx prima

001?0??????????????0001???1???0?????0000??????00??110???????????00??00?????????00
11001010?00??01??100????0?0?0????00?1100100010?????00000000?10??0021001000020?????
?0000102?000?12?0?1?00?0??000?0?010000000000?011??0000??000?0??00?0?000??00000000?
?1?00?0???0?????00010000??1?????1?00?000?20??????0??0000100?0?000010?0?00000????00?
0???0001?00100?0?0000????

Compsognathus longipes

?01?0?????????????????????1???????00000?????11???????00?????????0000000??1?????00
01001010100??01??00?????0?0?????000???0010001??????0?000000?10??002??000?0?????????
0000100?000?01?0??????????00?0???000000000001100?00????0?????00?0?0010000000000?0?
?00?0???0?1???00010000????????1?00?000?????????0??0?1???0???????????????00????????0???
?001000?00?0?0?00????

Buitreraptor gonzalozorum

?0110???????????????00001011?00??????????100?10??0011?0?????????00??001??????????
1210??100?0010111100111?010???100011012?121???????01101111010?1?10??0??????11?101??
1122???01???1?120???11?0??0??1?011?0?1110000????????1????????2101000110000?00???1???
?????????????00??001102??0??0??01???400??????1?????????00000????00?0??00?????????????0
01???0???0??000????

Unenlagia comahuensis

???
??????????????????11121112?10???1????1?????????????0??1001?????????????01111?1011111002
020110?121202011?10000??0?000??111100000????0??11????0??210110?110?????????????????
??0?????????????????????0????1?0400??0????????????????00?01??1?000000??1?0?????0001?0
00?0?0?????????

Shanag ashile

???????????????????1??1010110??????????????????????????????????????00??001??????????0
1001011???
???0????0????????????????10100001???00????????
????????00???0
???????

Mahakala omnogovae

???????????????101???????????????????????1?00?????????0?001?????????????????????????
??????????????????????????1?100011012?1?????????????????????11101000??00?211?11?2?????
??????????????111100????0?010?01101?0?0?0??00??11???????0000?????00????????0?????????
?11??????????????????????0??????????????1?????????00?000010??????000????000?0?0001?0?0?
00?????0????

Falcarius utahensis

??11?11101001111110?????1???????????????000??????001100000??????100?000??0?????0
?0010100011???0?011101011011?0112000110??0000??????000?01000010010010000100?01100
1?20120300010100100010000010001000110000000?00010??000?1??00?0?10000?001000?????
???0?001??101011??000111012112101111?000000100000111110001000020000000011?0000000

0011001?1000000000000?10?00000

Beipiaosaurus inexpectus

0?1??1??????????????????10?11????????????0
10010010???0???1??????????????000??0210?????????000???0????0?10010000?00011?0?1???10
220??0?00????0??00????00??????0000????0???0??0011????0?0??0????0010?0?????????0?????
??0??????1???11????????????00??0??????11??1?0000?00?0?001100????0?101?0?1??1???0?1???
01?000?????111?

Neimongosaurus yangi

??21????0???????????0?
00?00101??000111010112?0?1??2?0?1?02100???????0001010000??????????????111001?2???
?????????????????0??00????0??000000021?02????0?????30?0??0000?????0???????????0?????
???????0110??1121?211??10?1100?0200100101??????????2?11113210?????111110?111?????100
1?1?111??????10?

Erliansaurus bellamanus

???
?????????????????????????????????1???????????????0??????0?1?0????00000????1?0?1?????????
?????2??1????10?00?010001?0??00??????????00????????????????????0????????????????????
?????????????????????????0101100??11110001110????1???10???0?11111?011111111?0???1?11
1??????10?

Suzhousaurus megatherioides

???
???????????????01011201?0???0??1????0?????????001010000???????????000111021?2010?
2?0001002321010110 0?00??????????????????1???????????????00???010???????????????????
?2????????????????111?200???00120000101??????????2112222100211 1?111000????????????
?1???1??????001

Nothronychus graffami

???
???????????????01??120??1?1?00?110210?001?????00010?100?10???0?00000000??10?1?2010
22110010023 2101?1100000?10001100000021?01???00??????1?0??0000?010?00?????????????
?????1??????????????1??11?1?01110?1020?0??1?00?10?0???1211?22??2000111111 1101?111111
201??1?121??????10?

Nothronychus mckinleyi

????????2?0?1?0?0?0???????????????????????????????????00100???????????????????????0
??010??????000?1101?11?0?????????1???????0????????????100?100??0?????????????????0102
21?0?100??????????????010??1?0?????021??????00????????0?2??????10??????????????????012
101????????1121121111???1???10200000?001???????1???????????011???????????1111?????11?
?121?????????

Enigmosaurus mongoliensis

???
???111?21?20102221
00??0212101???00???010????????????????????2???
???????????????????0??????????????????????????2?1?22321?02100???????????????????????????
???????

Nanshiungosaurus brevispinus

??
???????1?????1?111???20??1??100111?21?2010???
10????2?210???00?????0????????????????????????
????????????12?1?1??0???????????????????????????21122222200?11???????????????????????????
????????

Therizinosaurus cheloniformis

??
??0010?0010????00200200?????????????????
???????????????????0??0?3100000002l?0????00??
??????????????????????0?1?211001?10110?01112?????????????????????????????11?01?????21?
????????

Hagryphus giganteus

??
??101000?2???????????????????
???0??
???????????????????????????????????001101000???
????

Dilong paradoxus

?0100???00000??????00010??1010000?0011?1000?1?010101????0???????0000000??00?00
?0010001010?11??0????01????0?0??????0??012?0??????????000?0011????????0000??00?1????
??001010??0?0?02??????0??????????????00100???00?00??0???00????0?0???00?000000000011
100?0???0????000001000?00??????????0003000?0??????00?01?0?0?00???????0?00?????0?????
????1?00?0000?0111????

Coelurus fragilis

??
???????0??00111100011000 0?????0?0000????????????0?0??0?10101?001?0??????????????????
00?????021201000000?0?0100110?0?0????0?01??00???0??????????0?0????0??????????????????
0????????????2002101000?00???000000000101?0?0?0???????????????000??00??0??0001000?0?
?0?000??????0??

Protarchaeopteryx robusta

?????????????????????0???0010
2?100??1?????????0????2??????????????1????????????????????????1010000??0??1???????????
?????????????????????????????00???000?0???????????????????????????????????????0??2??????
?????00?11?????????????11?????????????1??10?0??0?0?0???????????0???????????0001100?????
?1????0??

Yanornis martini

?01???1??????????????00??0????????????1?
?????0??????????????????????????????????2??????????????0??111?01??111?1??00?1?1????????????0
0?1?1?20?2???????????????????1???0?0???0?????????1???????????????1????????????01?????0??
????01?012??????????????10??00???0??????1?????0????0?????????00?????????????001?00?0???
?0???????

Jeholornis prima

??????0????????1??????????????0?????????????????????10?????????????10??0??????????1?2?
2????0???????????????????0?11???0221?0????01??????1?0?1111001??101000000l1?1??0??01??

??00?????22?2????????????????2??????????????????1????1?????0???1?????1??????????????????
0??????1??012?????????????010??00?????????????1???000?00?00????0?00????????????0001?10?
??0??0????0??

Jianchangosaurus yixianensis

??1?????????????????10010??1000??????0?00????????0000????????????010?110??????0?1?
00101000??0010001110?01?010?0???0??01???0?001??????00?0?0000100??0?0020000011?001?
2010120200?0001000100000??????0????0???0????000?0011?1?0000??0????0010?000000??0?
0001????????100110??01?11??0??1010??010?0?0?010001?0?0??00?0?00?001?0?000?010101??
0??????0?00000?0?00?0?111

Bissekty therizinosaurid

????????01??11???10???????????????????????000??????????00?????????21??1?0???????????
01001001????000111010112?????12?0??10???????????????01??0?0????????00?????????????????
???????????????01100000????0????00???21??????0?1??1?3??0?2?????????0??0??????0?001??0
?2??1??011???11211211?????0?????2??1??101?10??0??1??????????????????11100010?????1?0??
?1???1????0????

Lingyuanosaurus sihedangensis

??
????????????????????012??01???????0??????????????????????00?????????0??010?2?0??00???????
?????????????2?10??0????????????????????0??
?????????????????0??0????????????0202???????????10?20?20???????0111002??????????????1??????
???1100
